# Exploring the Effect of Pulsed Electric Fields on the Technological Properties of Chicken Meat

**DOI:** 10.3390/foods10020241

**Published:** 2021-01-25

**Authors:** Giulia Baldi, Fabio D’Elia, Francesca Soglia, Silvia Tappi, Massimiliano Petracci, Pietro Rocculi

**Affiliations:** 1 Department of Agricultural and Food Sciences, Alma Mater Studiorum, University of Bologna, Piazza Goidanich 60, 47521 Cesena, Italy; giulia.baldi4@unibo.it (G.B.); fabio.delia2@unibo.it (F.D.); francesca.soglia2@unibo.it (F.S.); silvia.tappi2@unibo.it (S.T.); 2 Interdepartmental Centre for Agri-Food Industrial Research, Alma Mater Studiorum, Campus of Food Science, University of Bologna, 47521 Cesena, Italy

**Keywords:** pulsed electric fields, chicken meat, meat quality, technological properties, meat proteins, water holding capacity

## Abstract

Pulsed electric field (PEF) is a non-thermal technology which is increasingly drawing the interest of the meat industry. This study aimed at evaluating the effect of PEF on the main technological properties of chicken meat, by investigating the role of the most relevant process parameters such as the number of pulses (150 vs. 300 and 450 vs. 600) and the electric field strength (0.60 vs. 1.20 kV/cm). Results indicated that PEF does not exert any effect on meat pH and just slightly affects lightness and yellowness. Low-intensity PEF treatments improved the water holding capacity of chicken meat by significantly (*p* < 0.001) reducing drip loss up to 28.5% during 4 days of refrigerated storage, without damaging proteins’ integrity and functionality. Moreover, from the analysis of the process parameters, it has been possible to highlight that increasing the number of pulses is more effective in reducing meat drip loss rather than doubling the electric field strengths. From an industrial point of view, the results of this explorative study suggested the potential of PEF to reduce the undesired liquid inside the package, thus improving consumer acceptance.

## 1. Introduction

Pulsed electric field (PEF) is a non-thermal technique that relies on the application of high-intensity electrical pulses for short duration times to biological tissues placed between two electrodes [1]. Albeit this technology was first adopted in the food industry about 50 years ago, PEF is still considered an emerging technology and is increasingly raising the interest of the scientific community due to its sustainability, environmental friendliness, and recent advancements in industrial applications [2,3]. PEF is applied as a single treatment or in combination with other technologies to synergistically enhance product quality, microbial stability, and process yields [4,5]. The abovementioned goals can be achieved through the modulation of several process parameters (e.g., electric field strength, number of pulses, time of treatment, etc.) leading to the electrical breakdown of cell membranes (i.e., electroporation) and resulting in the creation of pores, which work as conductive channels [6,7].

Although in the past 50 years several mechanisms about pore formation have been supposed [8,9,10], recent advancements in the field highlighted that the main mechanism of electroporation involves the development of aqueous pores in the lipid bilayer of the cell membrane, along with variations to individual membrane lipids and proteins, as suggested by Kotnik et al. [11]. In more detail, the same authors reported that pulsed electric fields trigger peroxidation to the membrane lipids, which causes deformations at the level of the lipid tails, thus increasing the permeability of the bilayer to water, ions, and other small molecules [12]. Depending on the electric field strength, the electroporation process can be either reversible or irreversible [13]. In the first case, the cell might be damaged but still able to fully recover, while in the second the membrane integrity is lost, causing an extensive leakage of intracellular content and, eventually, cell death [6,14]. Based on the reversibility of the process, PEF technology can have different applications in the food industry. Irreversible electroporation at high field strengths (e.g., 10–50 kV/cm) is particularly studied as an alternative to traditional thermal processing for microbial inactivation, with the advantage of minimally altering sensorial and nutritional characteristics of foods [1,14,15]. On the contrary, irreversible electroporation at lower field strength values (0.5–5 kV/cm), might be fruitfully exploited to enhance mass transfers during further technological processes such as drying, freezing, freeze-drying, and osmotic dehydration [8,16,17,18].

Having this in mind, the attraction towards the application of PEF in the meat industry has increased in recent years, as evidence grows on its ability to induce microstructural changes that might lead to the improvement of meat functional properties [19]. However, based on the available literature, a remarkable number of studies concerning the application of pulsed electric fields on pork and beef has been carried out over the past 20 years, while studies regarding the effect of PEF on poultry meat are still scarce. According to the last reports of the Organization for Economic Co-operation and Development (OECD), poultry is the most widely eaten type of meat worldwide [20]. With growing trends in further processing and increasing rates of broiler breast muscle abnormalities [21], meat technological properties (e.g., water holding capacity, emulsifying, and gelling properties, etc.) are gaining progressively more importance and the application of innovative technologies is actually considered as the most promising option for the improvement of such properties in poultry processing plants [1].

Considering these aspects, the knowledge concerning the feasibility of emerging technologies on chicken meat should be deepened. For this purpose, this study aimed at evaluating the implications of different PEF treatments on the main quality traits and the technological properties of chicken meat.

## 2. Materials and Methods

### 2.1. Experimental Design

#### 2.1.1. Trial 1

**Figure 1:**
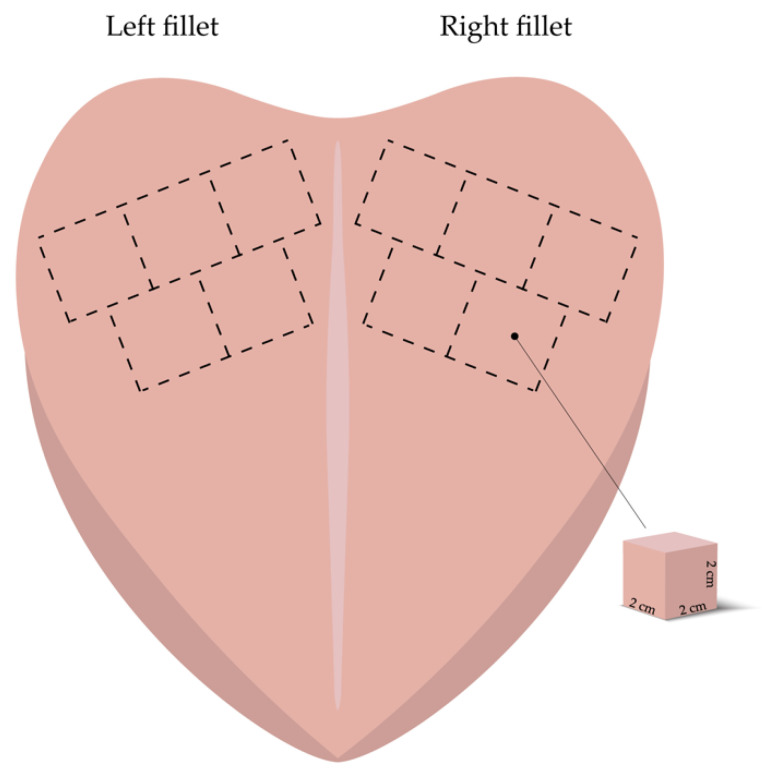
Sampling procedure adopted for the experiments. Meat samples were cut from the cranial position of each fillet following fibers direction (*n* = 10 meat samples/breast muscle).

Two separate trials were carried out as part of the experimental design of this study. For the first experiment, a total of five chicken breasts (*Pectoralis major* muscle) belonging to the same batch of broilers which were homogenous in weight, sex, and age were purchased at 24 h postmortem from a local market (Coop Italia Società Cooperativa, Cesena, Italy) and chilled at 4 °C ± 1. Breast muscles were cut to obtain both the left and right fillets. According to the scheme reported in Figure 1, with the aid of a scalpel, a total of five subsamples of 2 cm^3^ and weighing about 10 g were cut following fibers direction from the cranial position of each fillet (n = 10 subsamples/breast muscle), thus obtaining a total of 50 meat subsamples.

Samples thus obtained were randomly divided into five experimental groups according to PEF treatments (Table 1).

Treatment parameters were selected on the basis of previous studies that have reported that the electroporation process in animal tissues occurs when an electric field strength equal to or higher than 0.6 kV/cm is applied [4,19,22]. PEF treatments were performed using a laboratory-scale PEF system (Mod. S-P7500, Alintel, Pieve di Cento, Italy) delivering a maximum output current and voltage, respectively, of 60 A and 8 kV. The generator provides monopolar rectangular-shaped pulses and adjustable pulse width (5–20 µs), pulse frequency (50–500 Hz), and total treatment time (1–600 s). For this experiment, meat samples (dimension of 2 × 2 × 2 cm, weighing about 10 g) were placed in the treatment chamber (5 cm length × 5 cm width × 5 cm height) which consisted of two parallel stainless-steel electrodes (3 mm thick) with a 4.7 cm fixed gap. Output voltage and current were monitored using a PC-oscilloscope (Picoscope 2204a, Pico Technology, Cambridgeshire, UK). Meat samples were treated at room temperature in tap water, with an initial electrical conductivity of 246 ± 2 μS/cm at 18 °C, measured using an EC-meter (Mod. Basic 30, Crison, Barcelona, Spain), while control samples were not subjected at any treatment or exposed to water. Since it is generally held that the electric field distribution strongly depends upon the orientation of the electric field with respect to the muscle fibers [23], meat samples were placed between the electrodes in such a manner that the electric field was delivered perpendicularly to the muscle fibers to facilitate the eventual electroporation phenomenon. During PEF treatments, frequency (50 Hz), the distance between electrodes as well as the pulse width (20 µs) and the sample:water mass ratio (*w*/*w*, 1:10) were kept constant, while the electric field strength and the treatment time were modulated according to what is reported in Table 1. Temperature changes due to PEF treatments, measured with a temperature probe (mod. TESTO 445, Testo GmbH & Co, Milan, Italy), were negligible.

#### 2.1.2. Trial 2

As for the second trial, another batch of five chicken breasts (*Pectoralis major* muscle) homogenous in weight, sex, and age were purchased at 24 h postmortem from the same local market and collected as described for experiment 1. Meat samples were then randomly divided into five experimental groups according to PEF treatments (Table 2) and handled as described for the first experiment.

### 2.2. Analytic Determinations

#### 2.2.1. pH

The pH of meat samples was assessed following the procedure proposed by Jeacocke [24]. In detail, 2.5 g of manually minced meat were homogenized for 30 s at 13,500 rpm by UltraTurrax T25 Basic (IKA-Werke, Staufem im Breisgau, Germany) in 25 mL of a 5 mM sodium iodoacetate and 150 mM potassium chloride solution (pH 7.0). The pH of the homogenate was then assessed by a pH meter (mod. 3510, Jenway, Staffordshire, UK) previously calibrated at pH 4.0 and 7.0. The pH measurements of the PEF-treated samples were carried out immediately after the treatments.

#### 2.2.2. Color

Color (CIE L* = lightness, a* = redness, and b* = yellowness) of meat samples was assessed through a reflectance colorimeter (mod. Chroma Meter CR-400, Minolta, Milan, Italy) equipped with an illuminant source C and previously calibrated with a reference color standard ceramic tile. Due to the small dimension of the meat samples, color measurements were performed as a single measure before and immediately after the treatment, and data were expressed as the difference in lightness, redness, and yellowness (∆L*, ∆a*, ∆b*, respectively) following PEF process.

#### 2.2.3. Drip Loss

The term “drip loss” refers to the fluid released by raw meat through passive exudation during refrigerated storage [25]. Meat samples having homogenous weight (10 g) and dimensions (2 cm^3^) were individually weighed, placed in plastic boxes, and stored at 4 °C for 96 h. Then, each sample was cleaned from eventual superficial liquid accumulation using a paper towel and weighed to assess drip loss, calculated according to the formula:

Drip loss (%) = [(initial weight − weight after storage)/initial weight] × 100

Drip loss was assessed by repeatedly measuring the same sample after 24, 48, 72, and 96 h of refrigerated storage.

#### 2.2.4. Protein Solubility

Protein solubility was measured according to Warner et al. [26] with slight modifications. In detail, sarcoplasmic protein solubility was determined by homogenizing for 30 s at 13,500 rpm by UltraTurrax T25 Basic (IKA-Werke, Staufem im Breisgau, Germany) 1 g of meat in 10 mL of ice-cold 25 mM potassium phosphate buffer (pH 7.2). Homogenates were placed at 4 °C for 20 h and then centrifuged at 2600× *g* for 30 min. The protein concentration of the supernatant was measured by Bradford assay, using bovine serum albumin as standard. Total protein solubility (myofibrillar + sarcoplasmic) was similarly determined by homogenizing the same meat aliquot in 1.1 M KI and 0.1 M potassium phosphate buffer (pH 7.2). Myofibrillar protein solubility was then calculated as the difference between total and sarcoplasmic protein solubility.

#### 2.2.5. Protein Denaturation Enthalpy

Total protein denaturation enthalpy of control and treated samples was assessed through a differential scanning calorimeter (DSC) DSC Q20 (TA Instrument, Wetzlar, Germany), equipped with a low-temperature cooling unit Intercooler II (Perkin-Elmer Corporation, Waltham, MA, USA). Temperature and melting enthalpy calibrations were performed with ion exchanged distilled water (mp 0.0 °C) and indium (mp 156.6 °C), while the heat flow was calibrated using the heat of fusion of indium (ΔH = 28.71 J/g).

For the calibration, the same heating rate and dry nitrogen gas flux of 50 mL/min used for the analysis were applied. According to Baldi et al. [27], each muscle sample was weighed (about 25 mg) into a 50-µL aluminum pan, sealed hermetically, and then loaded into the DSC instrument at room temperature. The heating rate of DSC scans was 5 °C/min over a range of 20–90 °C. Empty aluminum pans were used as reference and for baseline corrections. Three replications for each meat sample were performed and results were elaborated through a PeakFit Software (SeaSolve Software Inc., Framingham, MA, USA).

#### 2.2.6. Statistical Analysis

Data were checked for normality by means of Shapiro–Wilk test (SAS Institute Cary, NC, USA), and variables characterized by a non-normal distribution were properly transformed. Then, data obtained from experiments 1 and 2 were separately analyzed using the one-way ANOVA option of the general linear model (GLM) procedure of SAS software (SAS Institute, Cary, NC, USA), considering PEF treatment as the main effect. Means were then separated using Tukey’s honestly multiple range test of the GLM procedure and considered significant at *p* < 0.05. Subsequently, differences among experimental groups were explored through preplanned orthogonal contrasts, which allow to involve linear combinations of group mean vectors instead of linear combinations of the variables. In more detail, multiple orthogonal contrasts analysis was performed in order to estimate the main effects of the treatment (control vs. treated), the electric field strength (low vs. high), as well as the number of pulses (150 vs. 300 and 450 vs. 600 for experiment 1 and 2, respectively) on the qualitative and technological traits of chicken meat.

## 3. Results

### 3.1. Experiment 1

Table 3 shows the results concerning the effects of PEF treatment on pH, color variations, and water holding capacity of chicken meat samples. The application of pulsed electric fields did not exert any effect on meat pH, regardless of both the electric field strength and the number of pulses. Regarding the color parameters, while both a* and b* were not affected by the PEF treatments, the lightness of meat (L*) was significantly modified, with T3 showing the greatest variations (3.38) in absolute terms. Intriguingly, the application of low electric field strengths (0.6 kV/cm) was associated with a significant increase in the lightness of meat if compared to higher electric field strengths (1.2 kV/cm) (*p* < 0.01), while the number of pulses did not exert any significant effect.

The most promising result is the one concerning meat water holding capacity, measured through the drip loss analysis. Aside from T1 group, meat samples subjected to PEF treatments showed significantly lower drip losses if compared to control after 24 h of refrigerated storage. Among the experimental groups, T4, characterized by the highest total specific energy input (1.19 kJ/kg, Table 1), exhibited the lowest values (1.85%). Overall, the analysis of preplanned orthogonal contrast evidenced that the application of PEF allowed to significantly reduce the loss of liquids from meat after 24 h of refrigerated storage (*p* < 0.001). In detail, both the electric field strength and the number of pulses exerted a remarkable effect on the drip loss (*p* < 0.05 and 0.001, respectively), however, an increase in the number of pulses (from 150 to 300) allowed to reduce drip losses to a greater extent if compared to the increment in the electric field strength (from 0.6 to 1.2 kV/cm). While no difference was detected at 48, 72, and 96 h of storage, total drip loss was found to be significantly different among the experimental groups (*p* < 0.05), as a direct consequence of the results obtained after the first sampling time. The application of PEF significantly reduced total drip loss of meat by 28.5% if compared to untreated samples (5.22 vs. 7.30%, *p* < 0.01). Intriguingly, total drip loss was found to be significantly affected by the number of pulses applied (*p* < 0.05), rather than the electric field strength.

Results concerning protein solubility and total denaturation enthalpy of chicken meat subjected to PEF treatments are shown in Table 4. The application of pulsed electric field did not exert any effect on the abovementioned parameters, regardless of neither the electric field strength nor the number of pulses applied.

### 3.2. Experiment 2

Since the outcomes obtained from experiment 1 suggested that the number of pulses exerts a predominant effect on meat technological properties rather than the electric field strength, a second trial was carried out in order to extend the experimental design by further increasing the number of pulses while keeping fixed the levels of electric field strength. Table 5 shows the results concerning pH, color variations, and water holding capacity of samples as affected by PEF treatments. Similarly to what was observed in experiment 1, the application of PEF did not exert any effect on meat pH, regardless of both the electric field strength and the number of pulses applied. Regarding color parameters, meat redness (a*) was not modified after any of the selected treatments, while both L* and b* parameters significantly varied after the application of PEF (*p* < 0.05 and 0.001, respectively). In more detail, meat samples subjected to T7 and T8 showed the most relevant variations of L* parameter (+2.60 and +2.34, respectively). As for b*, T5 was associated with a significant decrease in the yellowness of meat (−1.79), while no differences were detected among the other experimental groups, which shared increased b* values. The analysis of preplanned orthogonal contrast evidenced that a higher number of pulses (600 vs. 450) led to a remarkable increase of both L* and b* parameters (*p* < 0.01 and 0.001, respectively), while the application of low electric field strengths (0.6 kV/cm) was associated to a significant decrease in b* values (*p* < 0.01).

According to what was found in experiment 1, the application of PEF significantly affected the water holding capacity of meat samples after 24 h of refrigerated storage (*p* < 0.001). In more detail, meat samples subjected to T7 and T8 showed significantly lower drip loss values if compared to the other experimental groups (*p* < 0.05), while T5 and T6 exhibited average values analogous to those detected for untreated samples. Overall, orthogonal planned contrast analysis showed that both the electric field strength and the number of pulses exerted significant effects on the drip loss of meat (*p* < 0.05 and 0.001, respectively). However, an increase in the number of pulses (from 450 to 600) allowed to reduce drip loss values of almost the double if compared to what was observed for the increment in the electric field strength (from 0.6 to 1.2 kV/cm) (31.0% vs. 15.4%). While no differences were detected at 48, 72, and 96 h of storage, total drip loss was found to be significantly different among the experimental groups (*p* < 0.01), as a consequence of the results detected at 24 h. Overall, PEF-treated samples exhibited an 11.5% reduction in drip loss values (*p* < 0.05) if compared to their untreated counterparts. In more detail, the application of a higher number of pulses (600 instead of 450) allowed to obtain a significant reduction (*p* < 0.01) in the total amount of liquids lost from meat during the refrigerated storage.

Results concerning protein solubility and total protein denaturation enthalpy of chicken meat subjected to PEF treatments are shown in Table 6. The application of PEF treatments did not exert any effect on the solubility of proteins, with the only exception of the sarcoplasmic fraction solubility that was found to be significantly higher in samples subjected to T8 (*p* < 0.05). Furthermore, the analysis of preplanned contrasts allowed to evidence that the application of 600 pulses led to a significant increase in the solubility of sarcoplasmic proteins, which improved by about 12% if compared to 450 pulses (*p* < 0.01).

Regarding total protein denaturation enthalpy, the application of pulsed electric fields did not exert any significant effect, regardless of neither the electric field strength nor the number of pulses applied.

## 4. Discussion

The application of PEF is drawing the interest of the meat industry thanks to its ability to accelerate mass transfers during drying and brining, improve tenderization, enhance the micro diffusion of water-binding agents and increase meat safety [4]. However, the utilization of PEF to improve the technological properties of chicken meat is still an unexplored field. Overall data evidenced that PEF treatments tested within the experiments do not affect meat pH, besides the number of pulses and the electric field strength applied. This result corroborates what was found by Khan et al. [28], where the application of pulsed electric fields at both 0.30 and 1.25 kV/cm did not change the pH of chicken breast muscles. Similar results were also observed on beef [22,29]. However, the application of PEF with high total specific energy input (>150 kJ/kg) was found to be associated with a significant decrease in muscular pH values, due to a modification in meat conductivity caused by the leakage of intracellular liquids following cellular electroporation [30]. Considering that the highest total energy input achieved within this study was 2.42 kJ/kg (Table 1 and Table 2), it is reasonable to assume that the treatments performed in the experiments were not strong enough to induce cell breakdown and thus the leakage of cellular fluids.

Only few studies are available in the literature concerning the effect of PEF on meat color, which is known to be the main factor leading customers’ purchasing choices. A recent study carried out in the U.S. reported that consumers have a clear preference towards lighter colored poultry meats over darker ones, especially for breast meat [31]. The effect of PEF on meat color strongly depends upon the magnitude of the temperature increase during the treatment, which might alter the redox state of myoglobin [19]. Indeed, high-intensity treatments coupled with elevated numbers of repeats may increase samples’ temperature, thus promoting myoglobin oxidation and meat discoloration [5]. Data from the available literature showed that PEF treatments with high total specific energy input (>50 kJ/kg) caused a significant decrease of meat lightness [32], while milder settings did not affect color parameters of both beef and turkey meats [33,34]. Although low-intensity treatments (<5 kJ/kg) were performed within this study, PEF slightly changed meat lightness and yellowness, while redness was not affected. However, the trends observed for meat color variations were controversial between the two experiments and might be complex to interpret. Indeed, while in the first experiment a lower electric field strength and number of pulses were associated with an increase in meat lightness, in the second trial they were linked to a decrease in both lightness and yellowness of samples. Considering the low total specific energy input of the treatments (see Table 1 and Table 2) and the poor content of myoglobin in chicken meat [35], it is reasonable to assume that color variations were not likely due to an alteration of meat pigments caused by an increase in sample temperature during the process, but it might be linked to a possible redistribution of water within cellular compartments after the PEF application. Indeed, PEF might have favored the movement of water within cellular spaces, thus leading to a change in the refractive properties of the tissue [36]. Albeit further research should be performed to test this hypothesis, it is noteworthy to mention that meat color variations induced by PEF treatments in this study were negligible and probably not detectable by the human eye.

In muscular tissue, water is distributed in several compartments and can be present either as bound or free water: the first is tightly fastened to meat proteins, while the second is held between myofilaments and myofibrils, as well as outside the fibers [37]. There is growing evidence concerning the ability of PEF to modify the microstructure of meat through the formation of pores, thus suggesting its potential to influence the water holding capacity of meat by facilitating water movements [5]. Overall outcomes of this study indicated that low-intensity PEF treatments can significantly improve water holding capacity in chicken breast fillets by reducing the loss of liquids from meat from 13% up to 28.5% during 4 days of refrigerated storage. Moreover, in both the experiments, doubling the number of pulses reduced drip losses to a greater extent if compared to increasing the electric field strength. There are conflicting reports in the literature concerning the role of PEF on the water holding properties of meat; the divergences found among the studies are ascribable to the intrinsic properties of the muscle (i.e., amount of fat and connective tissue, pH, fiber diameter, muscle contractile state), the dimension and the initial moisture of the samples as well as PEF processing conditions (i.e., total specific energy input, number of pulses, frequency, etc.) [19]. It is generally believed that high-intensity PEF treatments leading to irreversible electroporation (i.e., irreversible electrical breakdown of cell membranes) are associated with an increase of drip loss from meat due to a number of mechanisms including protein denaturation, myofibril fragmentation as well as cell rupture and leakage of cell fluids into extracellular spaces [38]. Considering the utilization of low total specific energy inputs and short exposure times, it can be hypothesized that PEF treatments performed within this study have induced reversible cell electroporation, meaning that cell membranes momently modified their permeability without loosening their integrity [4,7]. Within this scenario, the reasons behind the remarkably reduced drip losses of meat following low-intensity PEF treatment might be different. The first hypothesis deals with a possible re-compartmentalization of moisture following cellular electroporation that might have facilitated water movements within extra- and intracellular compartments [1,5]. Indeed, the exposure of skeletal muscle tissue to a sufficiently high external electric field likely caused a rapid increase in membrane permeability (i.e., membrane electroporation) due to the formation of temporary pores in the phospholipid bilayer of the cell membranes [39]. The abovementioned pores are defined as “aqueous” since they are particularly hydrophilic; indeed, the application of the electric field causes the exposure of the polar heads of the membrane’s phospholipids allowing greater interaction with the water molecules [7]. Most theoretical works on electroporation suggest that following a period ranging from milliseconds up to few minutes after the field is removed, the pores reseal [39]. Given these compulsory details, it could be hypothesized a water re-compartmentalization following the temporary changes in membrane permeability that might have favored the transition of water from extra- to intramyofibrillar spaces within skeletal muscle tissue. Thus, water molecules penetrated into the lipid bilayer might be trapped into the pores, thus leading to a reduced water loss from meat. The second hypothesis is related to the potential of PEF to change the polarity of amino acids’ side chains, responsible for their hydrophilic or hydrophobic behavior [40]. In 1999, Yeom et al. [41] put forward the theory that high intensity PEF treatments are able to modify secondary and tertiary structures of proteins by increasing the content of β-sheets to the detriment of α-helices. This theory was further validated in 2008 by Zhao and Yang [3], who suggested that, as a direct consequence of proteins’ conformational changes, PEF might influence proteins’ hydrophobicity. Later on, the ability of PEF to break covalent bonds and generate new sorts of interactions within the peptide chains was reported by Poojary et al. [15]. Within this context, we could hypothesize that low intensity PEF treatments performed within this study may have induced proteins’ conformational changes which likely caused a modification of the attraction/repulsion interactions between polar and apolar amino acids, thereby enhancing the interactions between proteins and water molecules. However, based on the available literature and considering the different PEF intensity applied, it is not possible to recognize which of the two abovementioned mechanisms, taken individually or jointly, is responsible for the increased water holding capacity of meat following PEF. Thus, further studies must be carried out to deeply investigate water distribution in the muscle after the application of PEF. From a technological point of view, the potential of PEF to reduce water loss from meat during refrigerated storage might reduce the presence of undesired liquid inside the meat packages, improving consumer acceptance.

Within this experiment, the application of PEF did not exert any effect on the solubility of chicken meat protein fractions. Results in the literature concerning the role of PEF on muscular protein solubility are lacking, however, those available highlighted that the functional properties of proteins are drastically impaired as the intensity of PEF and the treatment time increase [42]. Accordingly, a recent study performed on proteins isolated from pale, soft, and exudative (PSE) chicken meat reported that the solubility of the myofibrillar fraction improved with the application of 18 kV/cm, while a further increase in the electric field strength was associated to a worsening of protein functionality, due to the occurrence of protein denaturation and aggregation [43]. Thus, since myofibrillar proteins are of great importance for meat technological properties (e.g., water holding capacity) [44], it is essential to modulate process parameters in order to avoid detrimental effects on meat proteins and their ability to hold water molecules. Considering the low electric field intensities applied within this study (<2.5 kV/cm), it might be reasonable that PEF did not negatively affect proteins solubility, thus preserving their functional properties. Moreover, DSC technique allowed to evidence that the application of PEF did not trigger protein denaturation processes, thus corroborating the outcomes concerning protein solubility which is generally considered as an indicator for protein denaturation level. However, considering the explorative approach of this study, further research should be carried out to validate these results and investigate the effective potential of this emerging technology to be applied in the poultry field.

## 5. Conclusions

This explorative study allowed to broaden the knowledge about the role of pulsed electric fields on the technological properties of chicken meat. Overall outcomes suggest that PEF treatments performed within the experiments improve the ability to retain water in meat without damaging proteins’ integrity and functionality. Based on these results, the application of PEF might represent a fruitful opportunity for the poultry industry to reduce the presence of undesired liquid inside the meat packages, improving consumer acceptance and meat yields. Further investigations must be carried out to deeply understand the mechanisms involved in the enhancement of water holding capacity after the application of PEF and establish whether this characteristic is preserved also during subsequent meat processing and cooking operations.

## Figures and Tables

**Table 1:** Pulsed electric field treatment parameters applied in experiment 1. Electric field strength and total specific energy input values are expressed as means ± standard deviation.

Treatments (T) ^1^	Electric Field Strength (kV/cm)	Energy Input ^2^ (kJ/kg)	Frequency (Hz)	Treatment Time (s)	Pulse Number ^3^	Pulse Width (µs)
Control	0	0	0	0	0	0
T1	0.60 ± 0.01	0.15 ± 0.01	50	3	150	20
T2	1.20 ± 0.02	0.59 ± 0.01	50	3	150	20
T3	0.60 ± 0.02	0.31 ± 0.01	50	6	300	20
T4	1.20 ± 0.03	1.19 ± 0.04	50	6	300	20

^1^*n* = 5 samples/treatment. ^2^ Calculated according to Raso et al. [16]. ^3^ Calculated by multiplying frequency (i.e., number of pulses/s) and total treatment time.

**Table 2:** Pulsed electric field treatment parameters applied in experiment 2. Electric field strength and total specific energy input values are expressed as means ± standard deviation.

Treatments (T) ^1^	Electric Field Strength (kV/cm)	Energy Input ^2^ (kJ/kg)	Frequency (Hz)	Total Treatment Time (s)	Pulse Number ^3^	Pulse Width (µs)
Control	0	0	0	0	0	0
T5	0.60 ± 0.02	0.45 ± 0.01	50	9	450	20
T6	1.20 ± 0.03	1.77 ± 0.05	50	9	450	20
T7	0.60 ± 0.02	0.66 ± 0.02	50	12	600	20
T8	1.20 ± 0.03	2.42 ± 0.09	50	12	600	20

^1^*n* = 5 samples/treatment. ^2^ Calculated according to Raso et al. [16]. ^3^ Calculated by multiplying frequency (i.e., number of pulses/s) and total treatment time.

**Table 3:** Average pH, color variation (∆L*, ∆a*, ∆b*), and water holding capacity (drip loss, %) of chicken meat samples subjected or not to pulsed electric field treatments. Data are expressed as least square means ± standard deviation.^a–d^ = means lacking a common letter significantly differ (*p* < 0.05). * = *p* < 0.05; ** = *p* < 0.01; *** = *p* < 0.001; n.s. = not significant.

				Parameters								
Treatment (T)	Electric Field Strength ^1^	Pulses *n*^o^	*n*	pH	∆L*	∆a*	∆b*	Drip Loss (%)				
								24 h	48 h	72 h	96 h	Total
Control	-	-	5	5.95 ± 0.07	-	-	-	3.95 ^a^ ± 0.54	0.79 ± 0.49	1.56 ± 0.55	1.19 ± 0.24	7.30 ^a^ ± 1.66
T1	Low	150	5	5.90 ± 0.08	2.34 ^ab^ ± 0.71	−0.40 ± 0.26	0.06 ± 0.69	3.42 ^ab^ ± 0.45	0.57 ± 0.28	1.33 ± 0.34	1.02 ± 0.28	6.21 ^ab^ ± 1.14
T2	High	150	5	5.93 ± 0.07	−0.46 ^bc^ ± 0.41	−0.44 ± 0.27	−0.56 ± 1.09	2.82 ^bc^ ± 0.41	0.50 ± 0.25	1.19 ± 0.40	1.00 ± 0.30	5.41 ^ab^ ± 1.25
T3	Low	300	5	5.95 ± 0.06	3.38 ^a^ ± 2.84	−0.22 ± 0.17	0.85 ± 1.18	2.25 ^cd^ ± 0.28	0.52 ± 0.20	1.10 ± 0.35	1.01 ± 0.37	4.79 ^b^ ± 1.05
T4	High	300	5	5.93 ± 0.06	−1.44^c^ ± 1.18	−0.47 ± 0.48	−0.39 ± 1.61	1.85 ^d^ ± 0.26	0.65 ± 0.17	1.04 ± 0.25	0.99 ± 0.26	4.46 ^b^ ± 0.84
*p*-Value				n.s.	**	n.s.	n.s.	***	n.s.	n.s.	n.s.	*
Planned contrast ^2^												
Control			5	5.95 ± 0.07	-	-	-	3.95 ± 0.54	0.79 ± 0.49	1.56 ± 0.55	1.19 ± 0.24	7.30 ± 1.66
Treated			20	5.93 ± 0.07	-	-	-	2.58 ± 0.69	0.56 ± 0.22	1.17± 0.33	1.01 ± 0.28	5.22 ± 1.20
*p*-Value				n.s.	-	-	-	***	n.s.	n.s.	n.s.	**
Low electric field strength			10	5.93 ± 0.07	2.87 ± 1.03	−0.32 ± 0.23	0.46 ± 1.01	2.83 ± 0.71	0.54 ± 0.23	1.21 ± 0.35	1.02 ± 0.31	5.50 ± 1.28
High electric field strength			10	5.93 ± 0.06	−1.01 ± 1.84	−0.45 ± 0.38	−0.46 ± 0.33	2.33 ± 0.60	0.58 ± 0.22	1.12 ± 0.33	1.00 ± 0.26	4.94 ± 1.12
*p*-Value				n.s.	**	n.s.	n.s.	*	n.s.	n.s.	n.s.	n.s.
150 pulses			10	5.91 ± 0.07	1.10 ± 0.79	−0.42 ± 0.25	−0.21 ± 0.89	3.12 ± 0.52	0.53 ± 0.25	1.26 ± 0.36	1.01 ± 0.28	5.81 ± 1.20
300 pulses			10	5.94 ± 0.06	0.97 ± 0.49	−0.34 ± 0.36	0.23 ± 0.49	2.05 ± 0.33	0.58 ± 0.19	1.07 ± 0.29	1.00 ± 0.30	4.62 ± 0.91
*p*-Value				n.s.	n.s.	n.s.	n.s.	***	n.s.	n.s.	n.s.	*

^1^ Low = 0.6 kV/cm; high = 1.2 kV/cm. ^2^ Control vs. treated (T1, T2, T3, T4); low electric field strength (T1, T3) vs. high electric field strength (T2, T4); 150 pulses (T1, T2) vs. 300 pulses (T3, T4).

**Table 4:** Average protein solubility (mg/g) and total protein denaturation enthalpy (∆H, J/g) of chicken meat samples subjected or not to pulsed electric field treatments. Data are expressed as least square means ± standard deviation. n.s. = not significant.

				Parameters			
Treatment (T)	Electric Field Strength ^1^	Pulses *n*^o^	*n*	Protein Solubility (mg/g)			Protein Denaturation Enthalpy (ΔH, J/g)
				Myofibrillar	Sarcoplasmic	Total	
Control	-	-	5	206.2 ± 32.3	164.8 ± 88.5	370.9 ± 103.9	2.66 ± 0.44
T1	Low	150	5	209.5 ± 47.3	161.1 ± 11.0	370.6 ± 52.3	2.90 ± 0.26
T2	High	150	5	229.6 ± 31.3	168.3 ± 5.60	397.9 ± 30.7	3.05 ± 0.27
T3	Low	300	5	229.5 ± 32.5	163.6 ± 26.5	393.1 ± 26.4	2.84 ± 0.20
T4	High	300	5	191.5 ± 19.3	204.5 ± 70.2	396.0 ± 33.3	2.54 ± 0.34
*p*-Value				n.s.	n.s.	n.s.	n.s.
Planned contrast ^2^							
Control			5	206.2 ± 27.4	164.8 ± 15.6	370.9 ± 33.6	2.66 ± 0.44
Treated			20	215.0 ± 35.2	174.4 ± 39.3	378.6 ± 38.7	2.83 ± 0.31
*p*-Value				n.s.	n.s.	n.s.	n.s.
Low electric field strength			10	219.5 ± 39.6	162.3 ± 19.2	381.8 ± 40.8	2.87 ± 0.22
High electric field strength			10	210.6 ± 31.7	186.4 ± 50.7	375.5 ± 38.4	2.80 ± 0.39
*p*-Value				n.s.	n.s.	n.s.	n.s.
150 pulses			10	219.5 ± 39.2	164.7 ± 9.06	384.2 ± 42.9	2.98 ± 0.26
300 pulses			10	210.5 ± 32.2	184.1 ± 54.5	373.0 ± 35.4	2.69 ± 0.30
*p*-Value				n.s.	n.s.	n.s.	n.s.

^1^ Low = 0.6 kV/cm; high = 1.2 kV/cm. ^2^ Control vs. treated (T1, T2, T3, T4); low electric field strength (T1, T3) vs. high electric field strength (T2, T4); 150 pulses (T1, T2) vs. 300 pulses (T3, T4).

**Table 5:** Average pH, color variations (∆L*, ∆a*, ∆b*), and water holding capacity (drip loss, %) of chicken meat samples subjected or not to pulsed electric field treatments. Data are expressed as least square means ± standard deviation. ^a,b^ = means lacking a common letter significantly differ (*p* < 0.05). * = *p* < 0.05; ** = *p* < 0.01; *** = *p* < 0.001; n.s. = not significant.

				Parameters								
Treatment (T)	Electric Field Strength ^1^	Pulses *n*^o^	*n*	pH	∆L*	∆a*	∆b*	Drip Loss (%)				
								24 h	48 h	72 h	96 h	Total
Control	-	-	5	5.95 ± 0.13	-	-	-	3.03 ^a^ ± 0.52	0.99 ± 0.28	1.12 ± 0.40	0.92 ± 0.32	6.08 ^ab^ ± 1.15
T5	Low	450	5	5.93 ± 0.12	0.03 ^b^ ± 0.84	−0.79 ± 0.67	−1.79 ^b^ ± 0.72	3.06 ^a^ ± 0.12	1.06 ± 0.19	1.11 ± 0.28	0.97 ± 0.15	6.20 ^a^ ± 0.47
T6	High	450	5	5.96 ± 0.11	0.64 ^ab^ ± 2.15	−0.26 ± 0.44	0.27 ^a^ ± 0.97	2.67 ^a^ ± 0.34	1.06 ± 0.16	0.94 ± 0.10	0.94 ± 0.09	5.60 ^ab^ ± 0.55
T7	Low	600	5	5.95 ± 0.14	2.60 ^a^ ± 1.35	−0.18 ± 0.07	0.80 ^a^ ± 0.83	2.17 ^b^ ± 0.14	0.99 ± 0.19	0.93 ± 0.10	0.91 ± 0.11	5.00 ^b^ ± 0.37
T8	High	600	5	5.94 ± 0.14	2.34 ^a^ ± 1.41	−0.60 ± 0.49	1.30 ^a^ ± 0.29	1.81 ^b^ ± 0.16	1.09 ± 0.35	0.88 ± 0.10	0.93 ± 0.11	4.71^b^ ± 0.58
*p*-Value				n.s.	*	n.s.	***	***	n.s.	n.s.	n.s.	**
Planned contrast ^2^												
Control			5	5.95 ± 0.12	-	-	-	3.03 ± 0.57	0.99 ± 0.23	1.12 ± 0.23	0.92 ± 0.16	6.08 ± 0.86
Treated			20	5.95 ± 0.12	-	-	-	2.42 ± 0.52	1.05 ± 0.22	0.97 ± 0.17	0.94 ± 0.11	5.38 ± 0.74
*p*-Value				n.s.	-	-	-	***	n.s.	n.s.	n.s.	*
Low electric field strength			10	5.94 ± 0.12	1.32 ± 1.72	−0.48 ± 0.55	−0.50 ± 1.55	2.61 ± 0.49	1.03 ± 0.19	1.02 ± 0.22	0.94 ± 0.13	5.60 ± 0.75
High electric field strength			10	5.95 ± 0.12	1.49 ± 1.94	−0.43 ± 0.47	0.79 ± 0.86	2.24 ± 0.52	1.08 ± 0.26	0.91 ± 0.10	0.93 ± 0.09	5.16 ± 0.71
*p*-Value				n.s.	n.s.	n.s.	**	*	n.s.	n.s.	n.s.	n.s.
450 pulses			10	5.95 ± 0.11	0.33 ± 1.58	−0.53 ± 0.60	−0.76 ± 1.35	2.86 ± 0.32	1.06 ±0.17	1.02 ± 0.22	0.95 ± 0.12	5.90 ± 0.58
600 pulses			10	5.95 ± 0.13	2.47 ± 1.30	−0.39 ± 0.40	1.05 ± 0.65	1.99 ± 0.24	1.04 ± 0.27	0.91 ± 0.10	0.92 ± 0.11	4.86 ± 0.48
*p*-Value				n.s.	**	n.s.	***	***	n.s.	n.s.	n.s.	**

^1^ Low = 0.6 kV/cm; high = 1.2 kV/cm. ^2^ Control vs. treated (T5, T6, T7, T8); low electric field strength (T5, T7) vs. high electric field strength (T6, T8); 450 pulses (T5, T6) vs. 600 pulses (T7, T8).

**Table 6:** Average protein solubility (mg/g) and total protein denaturation enthalpy (∆H, J/g) of chicken meat samples subjected or not to pulsed electric field treatments. Data are expressed as least square means ± standard deviation. ^a,b^ = means lacking a common letter significantly differ (*p* < 0.05). * = *p* < 0.05; ** = *p* < 0.01; n.s. = not significant.

.				Parameters			
Treatment (T)	Electric Field Strength ^1^	Pulses *n*^o^	*n*	Protein Solubility (mg/g)			Protein Denaturation Enthalpy (ΔH, J/g)
				Myofibrillar	Sarcoplasmic	Total	
Control	-	-	5	180.3 ± 38.4	160.8 ^ab^ ± 7.62	341.1 ± 43.9	2.95 ± 0.35
T5	Low	450	5	150.5 ± 34.4	155.9 ^ab^ ± 8.94	306.4 ± 32.2	3.21 ± 0.29
T6	High	450	5	160.3 ± 46.7	150.1 ^b^ ± 10.3	310.4 ± 49.2	3.36 ± 0.74
T7	Low	600	5	143.1 ± 37.8	169.3 ^ab^ ± 15.2	312.4 ± 24.4	3.32 ± 0.25
T8	High	600	5	144.8 ± 24.3	175.9 ^a^ ± 20.4	304.7 ± 37.9	3.30 ± 0.16
*p*-Value				n.s.	*	n.s.	n.s.
Planned contrast ^2^							
Control			5	180.3 ± 38.4	160.8 ± 7.62	341.1 ± 43.9	2.95 ± 0.35
Treated			20	149.7 ± 34.4	162.8 ± 16.9	312.5 ± 34.2	3.30 ± 0.41
*p*-Value				n.s.	n.s.	n.s.	n.s.
Low electric field strength			10	146.8 ± 34.3	162.6 ± 13.7	309.4 ± 27.1	3.26 ± 0.25
High electric field strength			10	152.5 ± 36.1	163.0 ± 20.4	315.5 ± 41.6	3.34 ± 0.53
*p*-Value				n.s.	n.s.	n.s.	n.s.
450 pulses			10	155.4 ± 34.4	153.0 ± 16.9	308.4 ± 34.2	3.30 ± 0.41
600 pulses			10	143.9 ± 39.0	172.6 ± 9.56	316.5 ± 39.3	3.31 ± 0.56
*p*-Value				n.s	**	n.s.	n.s.

^1^ Low = 0.6 kV/cm; high = 1.2 kV/cm. ^2^ Control vs. treated (T5, T6, T7, T8); low electric field strength (T5, T7) vs. high electric field strength (T6, T8); 450 pulses (T5, T6) vs. 600 pulses (T7, T8).

## Data Availability

The data presented in this study are available on request from the corresponding authors.
